# Prevalence and Determinants of Multiple Forms of Malnutrition among Adults with Different Body Mass Index: A Population-Based Survey in the Philippines

**DOI:** 10.1155/2023/3182289

**Published:** 2023-05-26

**Authors:** Wan-Chen Hsu, Aileen R. de Juras, Susan C. Hu

**Affiliations:** ^1^Department of Public Health, College of Medicine, National Cheng Kung University, Tainan City 701, Taiwan; ^2^Institute of Human Nutrition and Food, College of Human Ecology, University of the Philippines Los Baños, Laguna 4031, Philippines

## Abstract

**Background:**

The multiple forms of malnutrition, including overnutrition, undernutrition, and diet-related noncommunicable diseases, are emerging crises in Asian countries. Past studies have focused more on malnutrition among overweight/obese individuals; however, limited research has examined chronic energy-deficient adults. Therefore, this study is aimed at investigating the prevalence and determinants of different forms of malnutrition among adults with different body mass index, using the Philippines as an example. Findings from this study will guide the development and implementation of public health nutrition programs and policies.

**Methods:**

A representative dataset from the 2013 Philippine National Nutrition Survey was used in the study. Adults aged ≥20 years (*n* = 16,826) were included in the analysis after excluding those with missing values. Six phenotypes of malnutrition were assessed, including three in overweight/obese adults (overweight/obese with metabolic syndrome; those with micronutrient deficiency–anemia, vitamin A deficiency, and iodine insufficiency; and those with both metabolic syndrome and micronutrient deficiency) and three in chronic energy-deficient (CED) adults (CED with either metabolic syndrome or micronutrient deficiency and with both metabolic syndrome and micronutrient deficiency). Sociodemographic and lifestyle factors were examined as the determinants of different forms of malnutrition, and multinomial logistic regression analyses were performed.

**Results:**

The prevalence of the six phenotypes of malnutrition ranged from 0.4% to 10.2%, where overweight/obese with metabolic syndrome was the most predominant type. The multinomial logistic regression models indicated that older age was the major risk factor across all phenotypes. Sex was associated with the outcomes in the overweight/obesity group, whereas employment status was correlated with CED adults. Furthermore, higher educational levels, being married, living in affluent households, and not smoking were protective factors for conditions related to CED but not overweight/obese individuals.

**Conclusion:**

Malnutrition in all its forms is a significant public health concern that must be understood and addressed. Policymakers should implement appropriate intervention programs to control these nutritional problems considering the specific risk factors for the adult population.

## 1. Introduction

The different forms of malnutrition are a worldwide crisis affecting numerous countries, especially those in Asia [1]. The 2020 Global Nutrition Report estimates that 87% of the 143 countries are confronted with high levels of at least two forms of malnutrition (3 countries, overweight and stunting; 28 countries, anemia and stunting; and 56 countries, anemia and overweight) [2]. Worse still, 26% of those countries have experienced the cooccurrence of childhood stunting, anemia among women of reproductive age, and overweight/obesity (Ow/Ob) among adult women. Regarding the metabolic risk factors, high blood pressure and diabetes affect 22.1% and 8.5% of the adult population, respectively. These nutritional burdens have marked differences by the physiological group and sociodemographic characteristics such as age, sex, education, and wealth [2].

The Philippines is not exempted from these adversities and is persistently challenged by the various forms of malnutrition [3, 4]. The recent data in the Philippines indicate a twofold increase in overweight and obesity among adults, from 16.6% to 37.2% during 1993-2018. Parallel to this is the rising prevalence of metabolic syndrome (MetS) components, including abdominal obesity, hypertension, high fasting blood glucose, and dyslipidemia. Moreover, poor body weight, anemia, and vitamin A deficiency continue to have public health significance [3, 4].

However, past studies have focused more on overweight/obese people in the Philippines, and only limited studies have been conducted on underweight adults (also called chronic energy deficiency (CED)) [5–7]. In addition, most researchers have only examined a single measure of adult nutritional status (i.e., obesity, diet-related noncommunicable diseases, or anemia) [5–7]. This approach may fail to capture the severity of the nutritional problems, given that some adults concurrently suffer from more than one disorder. The consequences of the conditions mentioned above during adulthood are vast, ranging from reduced labor productivity to increased risk of morbidity and mortality. Thus, using a nationally representative sample, this study is aimed at investigating the prevalence and determinants of the different forms of malnutrition in both Ow/Ob and CED adults in the Philippines.

## 2. Materials and Methods

### 2.1. Participants and Sampling Procedure

The 2013 National Nutrition Survey (NNS) data from the Philippines was used [8]. The NNS data are cross-sectional and nationally representative and are collected every five years. Briefly, the survey is aimed at evaluating Filipinos' food intake, nutrition, and health status. It utilized the 2003 Master Sample of the National Statistics Office and employed a multi-stage-stratified sampling design. Barangays, enumeration areas, and households were the sampling units in the first, second, and third stages [3, 9]. The NNS provides bases for the country's programs and nutrition and health improvement plans. Further details of the methodology have been published elsewhere [10].

**Figure 1:**
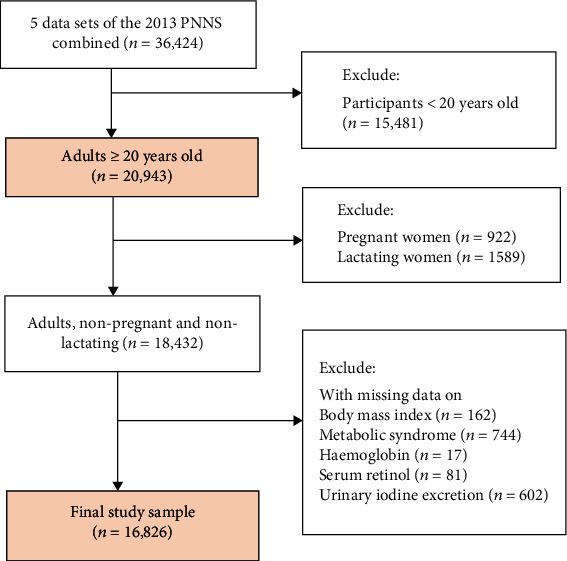
Flow diagram of participant selection. Missing data on metabolic syndrome was defined as no available data in at least 3 of the following: (1) waist circumference, (2) triglyceride, (3) HDL cholesterol, (4) blood pressure, and (5) fasting blood glucose.

The current analysis was limited to adults (≥20 years) with complete subject identification in five survey components: anthropometry, biochemical, clinical, and socioeconomic (individual and household). Pregnant and lactating women and those with missing values for the body mass index (BMI), MetS components, hemoglobin, serum retinol, and urinary iodine excretion (UIE) were excluded, accounting for 8.7% of the overall sample size. The final study sample included 16,826 adults (Figure 1).

### 2.2. Outcome Variables

The primary outcome variables were six phenotypes. The phenotypes included three categories for adults with overnutrition: (1) overweight/obese with metabolic syndrome (Ow/Ob+MetS), (2) overweight/obese with micronutrient deficiency (Ow/Ob+MnD), and (3) overweight/obese with both metabolic syndrome and micronutrient deficiency (Ow/Ob+MetS+MnD), and three categories for adults with undernutrition: (4) chronic energy deficiency with metabolic syndrome (CED+MetS), (5) chronic energy deficiency with micronutrient deficiency (CED+MnD), and (6) chronic energy deficiency with both metabolic syndrome and micronutrient deficiency (CED+MetS+MnD).

The anthropometry, biochemical, and clinical NNS datasets were used to classify individuals under the different phenotypes. Ow/Ob and CED were defined based on BMI derived from the weight and height measurements. Weight was measured by the Detecto™ platform beam balance weighing scale, while height was obtained using Seca™ microtoise [3]. The BMI of each participant was categorized according to the World Health Organization guidelines as CED (<18.5 kg/m^2^), Ow (25.0–29.9 kg/m^2^), and Ob (≥30.0 kg/m^2^) [11].

In this study, the National Cholesterol Education Program Adult Treatment Panel III clinical criteria were employed to assess MetS [12]. Based on the criteria, MetS was diagnosed if a person has any three of the following five criteria: (1) abdominal obesity (waist circumference > 102 cm in men or >88 cm in women); (2) dyslipidemia (triglyceride ≥ 150 mg/dL); (3) dyslipidemia, second criteria (HDL cholesterol < 40 mg/dL in men or <50 mg/dL in women); (4) hypertension (blood pressure ≥ 130/85 mmHg); and (5) hyperglycemia (fasting blood glucose ≥ 100 mg/dL). In addition, waist circumference was measured with a calibrated tape measure at the midpoint between the lowest rib and tip of the hip bone while the participants were standing and breathing normally. Blood pressure readings were performed with a calibrated nonmercurial sphygmomanometer (A&D Um-101™) and stethoscope on the right arm of seated participants after resting for a minimum of five minutes. The systolic and diastolic blood pressures were taken twice with two-minute intervals between the first and second measurements. Fasting venous blood samples were drawn from the participants for glucose and lipid assessments and analyzed through the enzymatic colorimetric method using the Roche COBAS Integra and Hitachi 912 clinical laboratory analyzer [3].

Furthermore, micronutrient deficiency (MnD) was characterized by anemia, vitamin A deficiency, and iodine insufficiency. Venous blood samples and urine samples were utilized to evaluate these conditions. Anemia was determined by measuring hemoglobin in the blood using a portable spectrophotometer [3, 13], where a hemoglobin value of <13 g/dL (males) or <12 g/dL (females) indicated anemia [14]. Vitamin A deficiency was determined from serum retinol levels using high-performance liquid chromatography [3, 15] and was defined as serum retinol < 10 *μ*g/dL [16]. Iodine insufficiency was assessed by measuring iodine excretion in the urine using the acid digestion/colorimetric method [3, 17]. The cut-off used for iodine insufficiency was UIE < 50 *μ*g/dL [18]. Healthy adults with normal weight (i.e., without MetS and MnD and BMI equivalent to 18.5–24.9 kg/m^2^) served as the reference group.

### 2.3. Explanatory Variables

The explanatory variables in this study were identified based on the literature review and information available in the NNS datasets. The individual-level sociodemographic factors included sex (male or female), age (20–39, 40–59, and ≥60 years), educational levels (highest level completed), marital status (single, married/with a partner, and others or those who were widowed/separated/divorced), and employment status (whether employed or not). The household-level sociodemographic factors covered the household size and wealth quintile. Household size was created from the socioeconomic dataset and categorized as 1–3, 4–6, and ≥7. Wealth status was divided into five groups (poorest, poor, middle, rich, or richest). Lifestyle factors, including smoking (current smoker or not), alcohol consumption (current drinker or not), and physical activity (low or high), were also controlled in the analysis. All variables were collected using standardized interviewer-administered questionnaires [3].

The study was conducted according to the guidelines in the Declaration of Helsinki and certified for exemption by the Human Research Ethics Committee of National Cheng Kung University, Tainan City, Taiwan (HREC No. 110–280).

### 2.4. Statistical Analysis

Descriptive statistics were used to summarize the weighted percentage of the participants' characteristics and outcome variables. A chi-square test was utilized to identify differences in the percentages obtained for the sociodemographic and lifestyle variables according to BMI. A bivariate analysis (chi-square tests) was also carried out to analyze factors associated with malnutrition phenotypes. Multinomial logistic regression models were performed on adults in the overnutrition and undernutrition groups. Healthy adults with normal weight were used as the reference group. The results were reported as odds ratios (OR) and 95% confidence intervals (95% CI). If the 95% CI does not include 1.0, it means statistically significant. Variance inflation factors (all < 2) were evaluated to verify the multicollinearity in the explanatory variables before running the models. All analyses considered the sampling design and survey weights and were performed using R software version 4.0.3 (R Foundation for Statistical Computing, Vienna, Austria).

## 3. Results

### 3.1. Participants' Characteristics

Table 1 describes the sociodemographic characteristics and lifestyle factors of the 16,826 valid adults (8428 men and 8398 nonpregnant and nonlactating women) included in this study. Compared to the average of the total sample size, the prevalence of Ow/Ob was higher among females (55.8%), middle-aged adults (47.8%), married individuals (75.8%), and noncurrent smokers (80.5%). In contrast, older adults (25.5%) with an elementary school education or less (42.0%), unemployed (49.4%), and noncurrent drinkers (54.7%) had a higher CED prevalence. Regarding wealth status, the prevalence of Ow/Ob increased with the quintile, ranging from 9.5% in the poorest to 31.1% in the richest households. Contrariwise, the prevalence of CED decreased from the poorest to the richest households (27.0% to 12.1%). The median household size was four, with no differences noted across BMI categories.

Table 2 shows that 29.6% of the adult population were Ow/Ob, 59.3% had normal weight, and 11.1% had CED. MetS was present in approximately one-quarter of the adults (24.6%) and was highest among those who were Ow/Ob (47.2%). In terms of MnD, 28.5% were either anemic, vitamin A-deficient, or iodine-insufficient and were more prevalent among adults who were CED (36.4%). The prevalence of both MetS and MnD was 7.5% and mainly affected adults who were Ow/Ob (12.7%).

### 3.2. Phenotypes of Malnutrition

The prevalence of the different phenotypes of malnutrition is shown in Table 3. Over one-third of the participants were healthy and with normal weight (35.5%), and nearly 40% only had a single form of malnutrition or MetS. Among the Ow/Ob adults, 1 in every 10 had MetS (10.2%), while a few had MnD (3.3%) and MetS+MnD (3.8%). CED was a concern in only a small percentage of these adults (0.4% for both MetS and MetS+MnD and 3.7% for MnD), and 3.3% were normal weight along with MetS+MnD.

In the bivariate analysis, the prevalence of the three conditions related to overnutrition (coexistence of Ow/Ob with MetS, MnD, and MetS+MnD) was higher among females, those 40–59 years old, those who were married, the richest quintile, noncurrent smokers, and noncurrent drinkers. Educational levels and household size were associated with all phenotypes in varying degrees of prevalence. For example, higher-educated adults tend to be more Ow/Ob+MetS, especially those with college degrees (12.6%). Adults with fewer family members had a higher proportion of Ow/Ob and MetS+MnD (3.4% to 4.2%). In contrast, the prevalence for adults with undernutrition (coexistence of CED with MetS, MnD, and MetS+MnD) was higher among those aged ≥60 years old, without a spouse, with elementary education or lower, unemployed, living in small-sized households (1–3 members), from the poorest or poor wealth quintiles, current smokers, and noncurrent drinkers. No differences were found in the level of physical activity in any phenotype.

### 3.3. Factors Associated with Overnutrition

Table 4 shows the factors associated with the different phenotypes of malnutrition relative to normal-weight and healthy adults, using multinomial logistic regressions. In the case of adults with overnutrition, sex, age, marital status, and the wealth quintile were risk factors for all three phenotypes. Women and married or widowed/separated/divorced adults were more likely to experience these conditions. Regarding the age group, the odds of any phenotype were higher among those 40–59 and ≥60 years old. Adults from the richest households had the highest risk of suffering from all phenotypes compared with those in the poorest quintile, with a significant dose-response relationship. In addition, having a high school and college education was related to MetS and MnD. Medium-sized households and not currently smoking were correlated solely with MnD. Notably, those from households with 4–6 members had a lower likelihood of MnD, and this was the only protective factor for Ow/Ob adults.

### 3.4. Factors Associated with Undernutrition

Table 4 also indicates that age was the common determinant of the three phenotypes for adults with undernutrition, while other variables had mixed effects on at least one phenotype. The odds of having any form of undernutrition and MetS were significantly higher among the older age group (≥60 years) as compared to the younger age group (20–39 years). Also, women, unemployed adults, and noncurrent drinkers were more likely to experience MnD. The remaining variables were protective factors for different phenotypes, including a college education or higher, married, big households (≥7 members), the middle wealth quintile, and noncurrent smokers. Interestingly, the odds of having MnD declined with improvements in wealth status.

## 4. Discussion

The results of this national study demonstrate the coexistence of Ow/Ob or CED alone or in combination with nutritional deficiency among adults ≥ 20 years old in the Philippines. The most predominant phenotype was Ow/Ob+MetS (10.2%). The other phenotypes were similar at approximately 3% (Ow/Ob+MnD, Ow/Ob+MetS+MnD, and CED+MnD) and 0.4% (CED+MetS and CED+MetS+MnD). Given the limited studies presenting the cooccurrence conditions among adults, the prevalence of Ow/Ob+MnD was lower in this study compared with the figures reported in Burkina Faso (3.3% vs. 8.5%) [19, 20].

This study found that age was the significant risk factor across all malnutrition phenotypes. The odds of older adults (≥60 years) were 1.6 to 14.0 times higher than that of younger adults. This finding could be linked to the high prevalence of single forms of malnutrition (i.e., Ow/Ob, CED, and MnD) and MetS components among elderly Filipinos, which is consistent with national estimates and previous studies [3, 5, 6, 21]. The strong correlation between age and the different phenotypes demonstrated in the outcomes may have been driven by the interactions among biological and behavioral factors [22].

Women were more likely to suffer from all categories of overnutrition. The higher prevalence of the coexistence of Ow/Ob, MetS, and MnD among Filipino women was expected because they are at greater risk for these conditions [3]. Some predisposing factors for women to develop these nutritional disorders are their reproductive biology and body fat distribution. Besides the reasons described above, socioeconomic and environmental factors also play crucial roles [23, 24].

The analysis also showed that educational levels, marital status, employment status, household size, wealth quintile, smoking, and alcohol consumption had mixed effects on the outcomes. Adults with higher educational levels, married, living in households with better wealth status, and not currently smoking had a greater risk of having conditions related to overnutrition. Remarkably, all these factors had an inverse relationship with the categories for adults with undernutrition.

Achieving a college degree or higher was a risk factor for the two phenotypes of adults with overnutrition. The opposite was true among adults with undernutrition, i.e., a higher level of education was a protective factor. This may partly be explained by the knowledge and skills gained from studying that enable individuals to make positive or negative choices about their diet, physical activity, and lifestyle [25]. Being married or living with a partner was also associated with the different phenotypes. The occurrence of stressors, perceptions of attractiveness, availability of resources, and the presence of a support system are posited to affect how marital status affects health outcomes [26, 27].

Employment was only associated with undernutrition, as CED+MnD was higher among unemployed adults. It is common in the Philippines to reside in urban areas with more job opportunities [28]. Consequently, these areas' physical and food environments may contribute to poor nutritional status. This finding also aligns with a past study wherein CED and anemia were more prevalent in certain occupational groups [5]. Larger household size was a protective factor for two phenotypes of malnutrition. This could have been due to changes in food intake quantity and quality with increasing family size, as supported by the national dietary survey results [3].

The influence of household wealth status on the study outcomes varied. Among the Ow/Ob adults, the rich and richest quintiles were related to all three phenotypes that could be attributed to the obesogenic effect of household wealth as it improves [29]. This result corresponds with previous research in India wherein the wealth index was a determinant of being overweight as well as MetS and anemia [30]. On the other hand, household wealth was protective for two phenotypes for adults experiencing undernutrition. The advancement in wealth status possibly had a lesser effect since most adults experiencing CED belonged to the poorest quintile. Additionally, this observation concurred with the study of Angeles-Agdeppa et al., wherein diet adequacy and diversity in Filipino households were similar across quintiles [31].

Furthermore, this study found that adults not currently smoking had greater susceptibility to Ow/Ob + MnD. It should be noted that the nonsmokers in this study included adults who had never smoked or were former smokers. Hence, it is probable that the relationship observed was for adults who had quit smoking. Evidence suggests that smoking cessation may lead to overnutrition and MnD through increased energy intake, inflammatory reactions, and oxidative stress [32–34]. Conversely, nonsmokers had a lower risk for CED+MnD and CED+MetS+MnD. This was ascribed to the clustering of healthy behaviors. As seen in this study, the prevalence of CED was higher among those who were not current smokers, those who were not current alcohol drinkers, and those who engaged in high levels of physical activity. However, the results on alcohol consumption warrant careful interpretation since the consumption of alcoholic beverages could alter nutrient metabolism and absorption, which could lead to malnutrition and MetS [35–37].

This study has two merits: the large sample size and the use of biochemical markers for the MetS and MnD assessment. However, it also has some limitations. First, the dataset did not include nonnutritional factors, such as disease history, medication use, and presence of infection, which might have provided more information on the determinants of malnutrition. Second, the lifestyle information was based on self-reporting, and certain behaviors may have been under- or overreported. Third, the exclusion of missing data may have introduced some bias. Lastly, the cross-sectional study design does not infer causality between risk factors and the development of overnutrition and undernutrition.

## 5. Conclusions

Our findings indicated that the cooccurrence of multiple forms of malnutrition among adults in the Philippines is a significant public health concern. Older age was the strongest risk factor in all phenotypes. In addition, being a woman was correlated with the categories for overnutrition, while being unemployed was associated with undernutrition. On the other hand, higher education, marriage, better-off households, and nonsmokers were protective factors related to undernutrition but not overnutrition. These results significantly contribute to understanding the different phenotypes of malnutrition and their potential determinants.

Therefore, public health policies and interventions are essential to address these threats from both ends of the spectrum. It highlights the importance of having adequate nutrition and health programs that consider socioeconomic status, for example, focusing on women who are overweight/obese in wealthy households and older adults who are underweight and unemployed. In addition, a healthy, sustainable food system and an increased investment in healthcare services are equally essential to improve malnutrition in all its forms.

## Figures and Tables

**Table 1:** Characteristics of participants according to the body mass index by chi-square tests^∗^.

Variables^†^	Total (*n* = 16,826) (%)	Overweight/obese (*n* = 4,768) (%)	Normal weight (*n* = 10,069) (%)	Chronic energy deficiency^‡^ (*n* = 1,989) (%)	*p* value
Sex					<0.001
Male	50.0	44.2	53.3	47.4	
Female	50.0	55.8	46.7	52.6	
Age group					<0.001
20–39 years	45.8	39.3	49.1	46.2	
40–59 years	38.9	47.8	36.4	28.3	
≥60 years	15.3	12.9	14.5	25.5	
Educational levels					<0.001
≤Elementary	31.9	23.9	33.9	42.0	
High school	38.3	39.6	38.2	35.6	
≥College	29.8	36.5	27.9	22.3	
Marital status					<0.001
Single	23.2	15.7	25.2	32.2	
Married	67.2	75.8	65.7	52.3	
Others	9.6	8.5	9.1	15.5	
Employment status					<0.001
Employed	60.1	61.3	61.2	50.6	
Unemployed	39.9	38.7	38.8	49.4	
Household size					0.084
1–3	33.2	33.7	32.7	34.4	
4–6	44.5	45.4	44.5	41.6	
≥7	22.3	20.8	22.8	24.0	
Wealth quintile					<0.001
Poorest	17.3	9.5	19.4	27.0	
Poor	19.3	15.4	20.5	23.6	
Middle	20.6	19.3	21.2	21.2	
Rich	21.2	24.7	20.4	16.1	
Richest	21.5	31.1	18.5	12.1	
Current smoker					<0.001
Yes	27.1	19.5	30.0	32.3	
No	72.9	80.5	70.0	67.7	
Current alcohol drinker					0.001
Yes	51.6	50.1	53.6	45.3	
No	48.4	49.9	46.4	54.7	
Physical activity					0.005
Low	43.3	45.6	41.6	46.5	
High	56.7	54.4	58.4	53.5	

^∗^Values are weighted percentages (%). ^†^Variables with missing observations: educational levels (*n* = 71), smoking and drinking status (*n* = 971), and physical activity classification (*n* = 1160). ^‡^Chronic energy deficiency (CED) also refers to being underweight and is defined as a body mass index < 18.5 kg/m^2^.

**Table 2:** Prevalence of metabolic syndrome, micronutrient deficiency, and their combination according to the body mass index by chi-square tests^∗^.

Variables^†^	Total (*n* = 16,826) (%)	Overweight/obese (*n* = 4,768) (%)	Normal weight (*n* = 10,069) (%)	Chronic energy deficiency^‡^ (*n* = 1,989) (%)	*p* value
MetS components (MetS)					
Abdominal obesity	11.9	36.1	1.9	0.2	<0.001
Hyperglycemia	20.3	29.6	16.9	13.6	<0.001
Hypertension	32.9	46.3	28.1	23.2	<0.001
High triglycerides	39.6	55.0	35.3	21.2	<0.001
Low HDL cholesterol	70.3	79.3	67.7	60.8	<0.001
MetS (≥3 of the above factors)	24.6	47.2	16.5	7.6	<0.001
Micronutrient deficiency (MnD)					
Anemia (yes)	6.5	4.7	6.4	11.8	<0.001
Vitamin A deficiency (yes)	0.1	0.1	0.1	0.2	0.368
Iodine insufficiency (yes)	23.8	20.2	24.8	28.6	<0.001
MnD (≥1 type)	28.5	23.8	29.3	36.4	<0.001
MetS+MnD	7.5	12.7	5.6	3.6	<0.001

MetS: metabolic syndrome; HDL: high-density lipoprotein; MnD: micronutrient deficiency. ^∗^Values are weighted percentages (%). ^†^Metabolic syndrome component variables with missing observation as follows: abdominal obesity (*n* = 254), hyperglycemia (*n* = 457), hypertension (*n* = 65), and high triglycerides and low HDL cholesterol (*n* = 16). ^‡^Chronic energy deficiency (CED) also refers to being underweight and is defined as a body mass index < 18.5 kg/m^2^.

**Table 3:** Prevalence of different phenotypes of malnutrition based on related determinants by chi-square tests^∗^ (*n* = 16,826).

Variables		Healthy and Nw (%)	Ow/Ob/CED/MetS/MnD (%)	Nw	Ow/Ob			CED			*p* value
				MetS+MnD (%)	MetS (%)	MnD (%)	MetS+MnD (%)	MetS (%)	MnD (%)	MetS+MnD (%)	
Size of the sample (*n*) (weighted %)		5685 (35.5)	6624 (39.5)	680 (3.3)	1709 (10.2)	544 (3.3)	689 (3.8)	93 (0.4)	712 (3.7)	90 (0.4)	—

Sex	Male	38.7	40.0	3.1	9.0	2.6	2.6	0.5	3.1	0.3	<0.001
	Female	32.2	38.9	3.5	11.4	3.9	4.9	0.4	4.2	0.5	

Age group	20–39 years	44.9	39.7	1.0	7.1	2.8	1.6	0.1	2.7	0.2	<0.001
	40–59 years	29.5	39.5	4.1	13.4	4.1	5.6	0.5	3.0	0.3	
	≥60 years	22.3	38.8	8.4	11.5	2.6	5.3	1.3	8.4	1.5	

Educational levels	≤Elementary	32.5	40.4	5.0	8.0	2.3	4.0	0.9	5.9	0.8	<0.001
	High school	37.2	38.9	2.8	10.2	3.8	3.6	0.3	3.0	0.3	
	≥College	36.4	39.3	2.2	12.6	3.7	3.6	0.1	2.1	0.0	

Marital status	Single	44.5	40.3	1.6	5.9	1.8	1.3	0.2	4.1	0.3	<0.001
	Married	33.7	39.5	3.3	11.7	3.8	4.4	0.5	2.8	0.3	
	Others	26.4	37.2	7.7	10.1	3.3	5.1	0.8	8.2	1.3	

Employment status	Employed	36.9	39.6	2.9	10.4	3.2	3.4	0.4	2.8	0.3	<0.001
	Unemployed	33.3	39.3	4.0	9.9	3.4	4.2	0.5	5.0	0.6	

Household size	1–3	33.5	38.6	4.2	10.4	3.6	4.2	0.5	4.3	0.7	<0.001
	4–6	35.3	40.3	3.1	10.8	3.2	3.6	0.4	3.1	0.3	
	≥7	38.7	39.2	2.5	8.8	2.9	3.4	0.4	3.8	0.2	

Wealth quintile	Poorest	36.5	42.8	3.2	4.7	2.2	2.5	0.8	6.7	0.6	<0.001
	Poor	36.0	40.2	3.7	7.5	3.0	3.6	0.8	4.5	0.6	
	Middle	36.2	39.3	3.8	9.1	3.0	4.1	0.3	3.8	0.4	
	Rich	35.7	37.4	3.4	12.9	3.9	3.9	0.3	2.4	0.2	
	Richest	33.2	38.3	2.5	15.4	4.1	4.4	0.1	1.6	0.2	

Current smoker	Yes	40.0	38.8	3.1	8.3	1.8	2.4	0.7	4.3	0.6	<0.001
	No	33.6	39.6	3.5	11.0	3.8	4.4	0.4	3.4	0.3	

Current drinker	Yes	39.1	38.8	2.5	10.0	3.1	3.1	0.4	2.7	0.3	<0.001
	No	31.4	40.0	4.4	10.5	3.4	4.6	0.5	4.7	0.5	

Physical activity	Low	34.6	39.2	3.6	11.0	3.1	3.9	0.4	3.9	0.4	0.232
	High	36.1	39.4	3.3	9.6	3.3	3.8	0.5	3.6	0.4	

Nw: normal weight; Ow/Ob: overweight or obese; CED: chronic energy deficiency; MetS: metabolic syndrome; MnD: micronutrient deficiency. ^∗^Values are weighted percentages (%).

**Table 4:** Factors associated with the different phenotypes of malnutrition among overweight/obese and chronic energy-deficient adults by multinomial logistic regression^∗^^,†^.

	Ow/Ob			CED		
	MetS (*n* = 1,709)	MnD (*n* = 544)	MetS+MnD (*n* = 689)	MetS (*n* = 93)	MnD (*n* = 712)	MetS+MnD (*n* = 90)
	OR (95% CI)	OR (95% CI)	OR (95% CI)	OR (95% CI)	OR (95% CI)	OR (95% CI)
Female (ref = male)	1.34 (1.14, 1.56)	1.34 (1.02, 1.76)	1.77 (1.37, 2.29)	1.14 (0.65, 2.01)	1.29 (1.01, 1.66)	1.98 (1.00, 3.90)
Age group (ref = 20–39)						
40–59 years	2.68 (2.21, 3.26)	1.98 (1.57, 2.49)	4.82 (3.67, 6.32)	3.94 (1.74, 8.92)	2.02 (1.52, 2.68)	1.98 (0.67, 5.86)
≥60 years	3.05 (2.43, 3.83)	1.64 (1.16, 2.32)	5.41 (3.83, 7.65)	14.25 (5.94, 34.19)	5.64 (4.10, 7.77)	10.20 (3.68, 28.29)
Educational levels						
≤Elementary (ref)						
High school	1.32 (1.12, 1.56)	1.76 (1.34, 2.32)	1.14 (0.90, 1.45)	0.55 (0.28, 1.05)	0.80 (0.63, 1.02)	0.73 (0.42, 1.28)
≥College	1.40 (1.07, 1.81)	1.67 (1.19, 2.33)	1.19 (0.88, 1.60)	0.31 (0.11, 0.89)	0.74 (0.54, 1.02)	0.11 (0.03, 0.42)
Marital status (ref = single)						
Married	1.77 (1.41, 2.23)	2.39 (1.70, 3.37)	2.09 (1.45, 3.00)	0.65 (0.30, 1.42)	0.45 (0.34, 0.60)	0.50 (0.18, 1.34)
Others	1.42 (1.05, 1.93)	2.19 (1.38, 3.47)	1.96 (1.31, 2.95)	0.75 (0.28, 1.99)	0.75 (0.52, 1.08)	0.80 (0.29, 2.17)
Employment status (ref = employed)						
Unemployed	0.98 (0.83, 1.14)	1.09 (0.86, 1.37)	1.12 (0.90, 1.38)	1.30 (0.71, 2.38)	1.80 (1.44, 2.25)	1.49 (0.79, 2.79)
Household size (ref = 1–3)						
4–6	0.96 (0.81, 1.12)	0.77 (0.61, 0.98)	0.82 (0.66, 1.01)	1.17 (0.70, 1.96)	0.94 (0.76, 1.16)	0.65 (0.37, 1.16)
≥7	0.82 (0.66, 1.00)	0.76 (0.57, 1.03)	0.91 (0.69, 1.19)	1.11 (0.58, 2.15)	1.09 (0.83, 1.44)	0.43 (0.19, 0.98)
Wealth quintile (ref = poorest)						
Poor	1.48 (1.10, 1.99)	1.33 (0.96, 1.84)	1.52 (1.04, 2.21)	1.19 (0.69, 2.04)	0.74 (0.56, 0.97)	1.33 (0.71, 2.50)
Middle	1.66 (1.24, 2.21)	1.19 (0.82, 1.73)	1.65 (1.12, 2.43)	0.48 (0.25, 0.92)	0.60 (0.45, 0.82)	1.03 (0.53, 2.00)
Rich	2.61 (1.93, 3.53)	1.64 (1.14, 2.36)	1.73 (1.15, 2.62)	0.48 (0.23, 1.03)	0.37 (0.26, 0.52)	0.50 (0.19, 1.30)
Richest	3.05 (2.19, 4.24)	1.82 (1.25, 2.65)	1.96 (1.28, 2.99)	0.40 (0.14, 1.21)	0.31 (0.21, 0.48)	0.89 (0.31, 2.58)
Nonsmoker (ref = smoker)	1.15 (0.96, 1.37)	2.07 (1.50, 2.85)	1.38 (0.97, 1.95)	0.69 (0.43, 1.10)	0.70 (0.56, 0.87)	0.56 (0.33, 0.96)
Nondrinker (ref = drinker)	0.96 (0.83, 1.13)	0.93 (0.73, 1.18)	1.05 (0.84, 1.31)	1.00 (0.58, 1.72)	1.66 (1.31, 2.10)	1.37 (0.77, 2.42)
Low physical activity (ref = high)	1.07 (0.91, 1.25)	0.87 (0.70, 1.08)	0.94 (0.76, 1.16)	1.05 (0.64, 1.73)	0.92 (0.75, 1.14)	0.82 (0.46, 1.46)

Ow/Ob: overweight or obese; CED: chronic energy deficiency; MetS: metabolic syndrome; MnD: micronutrient deficiency. ^∗^Values are odds ratios (OR) and 95% confidence intervals (95% CI). If the 95% CI does not include 1.0, it means statistically significant. ^†^All models were controlled for variables shown in the first column. Reference category: healthy and normal weight (*n* = 5685).

## Data Availability

The data is publicly available and can be found at http://enutrition.fnri.dost.gov.ph/site/home.php.
